# A Novel Dermaseptin Isolated from the Skin Secretion of *Phyllomedusa tarsius* and Its Cationicity-Enhanced Analogue Exhibiting Effective Antimicrobial and Anti-Proliferative Activities

**DOI:** 10.3390/biom9100628

**Published:** 2019-10-18

**Authors:** Miaoran Li, Xinping Xi, Chengbang Ma, Xiaoling Chen, Mei Zhou, James F. Burrows, Tianbao Chen, Lei Wang

**Affiliations:** School of Pharmacy, Queen’s University Belfast, Belfast BT9 7BL, UK; mli09@qub.ac.uk (M.L.); x.xi@qub.ac.uk (X.X.); x.chen@qub.ac.uk (X.C.); m.zhou@qub.ac.uk (M.Z.); j.burrows@qub.ac.uk (J.F.B.); t.chen@qub.ac.uk (T.C.); l.wang@qub.ac.uk (L.W.)

**Keywords:** antimicrobial peptide, frog skin secretion, dermaseptin

## Abstract

A novel dermaseptin peptide, dermaseptin-PT9 (DPT9), was isolated and identified from *Phyllomedusa tarsius* by the combination of molecular cloning and LC-MS analysis. Chemically synthesised DPT9 was broadly effective against the tested microorganisms through the disruption of cell membranes and showed weak haemolytic activity towards horse erythrocytes. It also exhibited anti-proliferative effect against various human cancer cells. Moreover, an analogue with enhanced cationicity, K^8, 23^-DPT9, in which Asp^8^ and Glu^23^ were substituted by lysine residues, had a markedly increased antimicrobial effect against all tested microorganisms and disrupted microbial cell membranes. This analogue also showed no haemolysis at its effective antimicrobial concentrations. In addition, K^8, 23^-DPT9 displayed an enhanced anti-proliferative effect against cancer cells, while displayed weak activity against the normal human cell line, HMEC-1.

## 1. Introduction

Antimicrobial peptides have gained much attention as novel antimicrobial agents. Most of them are cationic that they are able to kill the microbes through the electrostatic interaction with negatively charged lipid membrane of microorganisms, causing irreversible and potent membrane disruption and cellular dysfunction [1,2,3]. The selectivity and biological activity of these antimicrobial peptides is influenced by multiple physicochemical parameters. It is important to note that structural properties including charge, conformation, amphipathicity, hydrophobic moment, and hydrophobicity contribute to their mechanism of bacteria-killing action. Therefore, a better understanding of structure–activity relationships can contribute to the design of novel peptides with optimized potency [3,4].

Dermaseptins are a group of antimicrobial peptides that have been predominantly isolated from the frogs belonging to Phyllomedusinae subfamily, and they exhibit antimicrobial activity against an array of pathogens such as bacteria, yeasts, protozoa and filamentous fungi [5,6]. These peptides are a class of polycationic molecules that can adapt amphiphilic α-helical structures when interacting with membrane bilayers and their mechanism of action is associated with interaction, integration and subsequent lysis of cell membranes [7,8]. Previous research has demonstrated that dermaseptins permeate and disrupt the lipid bilayer of target cells via a “carpet” mechanism [9,10]. In addition, dermaseptins have been found to possess anti-proliferative activity and display different potency against various human cancer cells. These peptides could, therefore, provide new insights for the development of new anticancer agents [11,12,13,14,15].

Herein, a novel dermaseptin peptide was identified from the skin secretion of *Phyllomedusa tarsius* by the combination of cloning and mass spectrometry, namely dermaseptin-PT9 (DPT9). It demonstrates a high degree of similarity to the dermaseptin-5.1TR/5.2TR, while the biological functions have not been studied yet [16]. Therefore, antimicrobial, anti-proliferative and haemolytic activities were examined with synthesised DPT9 and its cationicity enhanced analogue to elucidate the influence of net positive charges on the biological functions.

## 2. Materials and Methods

### 2.1. Acquisition of Phyllomedusa tarsius Skin Secretion

Specimens of the *Phyllomedusa tarsius* (n = 3) were commercially purchased (PeruBiotech E.I.R.L, Huánuco, Peru) and maintained in our purpose-designed amphibian facility. The skin secretion was obtained by mild transdermal electrical stimulation and hand massaging [17]. The procedures were carried out as described previously [13]. The study was performed according to the guidelines in the UK Animal (Scientific Procedures) Act 1986, project license PPL 2694, issued by the Department of Health, Social Services and Public Safety, Northern Ireland. Procedures had been vetted by the Institutional Animal Care and Use Committee (IACUC) of Queen’s University Belfast and approved on 1 March, 2011.

### 2.2. “Shotgun” Cloning of DPT9 Precursor-Encoding cDNA from a Skin Secretion-Derived cDNA Library

The precursor-encoding cDNA of the peptide from was isolated from a skin secretion-derived cDNA library as in a previous study [12]. The 3′-RACE reactions employed a nested universal (NUP) primer (supplied with the kit) and a degenerate sense primer (5′-CCMRWCATGKCTTTCHTDAAGAAATCT-3′).

### 2.3. Isolation and Identification of DPT9 from Crude Skin Secretion

The process of isolating the mature peptide from crude skin secretion by reverse-phase (RP)-HPLC (Jupiter C-18 250 mm × 10 mm, Phenomenex, Macclesfield, UK) and the primary structure analysis of the novel peptide by tandem mass spectrometric (MS/MS) fragmentation sequencing used an LCQ-fleet electrospray ion-trap mass spectrometer (Thermo Fisher Scientific, San Francisco, CA, USA) as previously described [12].

### 2.4. Chemical Synthesis of DPT9 and K^8, 23^-DTP9

DPT9 and its analogue, K^8, 23^-DTP9 were chemically synthesized via solid-phase Fmoc chemistry using a Tribute automated peptide synthesizer (Protein Technologies, USA) with Rink amide resin. The synthesised peptides cleaved from resin by a cleavage cocktail containing trifluoroacetic acid (TFA), ethanedithiol (EDT), thioanisole and water (*v*/*v*/*v*/*v* = 94:2:2:2). The peptides were precipitated and washed by ice-cold diethyl ether, and further dissolved for lyophilisation. The final synthetic peptides were purified and identified by using RP-HPLC and matrix-assisted laser desorption/ionization, time of flight (MALDI-TOF) MS (Voyager DE, Perseptive Biosystems, Framingham, MA, USA), and then lyophilised as TFA salts.

### 2.5. Secondary Structure Analysis of DPT9 and K^8, 23^-DTP9

Physico-chemical properties of the peptides were predicted by Heliquest and the helical wheel plots of the secondary structures were obtained from the helical wheel projections [18]. The secondary structure of the synthesized peptide was determined using a JASCO J-815 circular dichroism (CD) spectrometer (JASCO Inc., Easton, MD, USA) as performed previously [19]. The result data were analyzed via the DICHROWEB webserver [20].

### 2.6. Antimicrobial and Antibiofilm Susceptibility Assay

The minimal inhibitory concentration (MIC) and minimum bactericidal concentration (MBC) of the synthetic peptides were assessed using different microorganisms including Gram-positive bacterium *Staphylococcus aureus* (*S. aureus*) (NCTC 10788), methicillin-resistant *Staphylococcus aureus* (MRSA) (NCTC12493), and *Enterococcus faecalis* (*E. faecalis*) (NCTC 12697); Gram-negative bacteria *Escherichia coli* (*E. coli*) (NCTC 10418), *Pseudomonas aeruginosa* (*P. aeruginosa*) (ATCC27853), and *Klebsiella pneumoniae* (*K. pneumoniae*) (ATCC 43816), and the yeast *Candida albicans* (*C. albicans*) (NCYC 1467). The determination of MICs and MBCs of the peptides was described previously [12].

Anti-biofilm activity of the peptides was assessed using the minimum biofilm inhibitory concentration (MBIC) and the minimum biofilm eradication concentration (MBEC) assays against *S. aureus*, MRSA and *E. coli* as previously performed [21].

Gentamicin (5 µg/mL), vancomycin (5 µg/mL) and amphotericin B (10 µg/mL) were employed as positive controls for Gram-negative bacteria, Gram-positive bacteria and *C. albicans*, respectively.

### 2.7. Haemolysis Assay

Defibrinated horse erythrocytes (TCS Biosciences Ltd., Buckingham, UK) were used to evaluate the haemolytic activity of the peptides. A total of 4% (*v*/*v*) erythrocytes were incubated with an equal volume of peptide solution in a range of final concentration from 1 to 512 μM in a 96 well plate as performed previously [12]. A total of 1% Triton X-100 and phosphate-buffered saline (PBS) were applied as the positive and negative controls, respectively.

### 2.8. Membrane Permeability Assay

Peptide solutions mixed with bacterial suspension reached final concentration of MIC, 2 × MIC and 4 × MIC for respective bacteria in a 96 well black plate. The cells were stained with 5 µM SYTOX green nucleic acid stain and the fluorescent intensity was measured using a Synergy HT plate reader (Biotech BioTek EL808, Winooski, VT, USA) as performed in previously [12]. The microbes treated by 70% isopropanol were used as positive control.

### 2.9. MTT Assay

The human neuronal glioblastoma cell lines, U251MG (ECACC-09063001), the human breast cancer cell lines MCF-7 (ATCC-HTB-22), the human pancreatic cancer cell lines, PANC-1, the non-small cell lung cancer cell line NCl-H157 (ATCC-CRL-5802) and the human prostate carcinoma cell line PC-3 (ATCC-CRL-1435) were selected to test the cytotoxicity of the peptides. The human microvascular endothelial cell line, HMEC-1 (ATCC-CRL-3243) was utilized to evaluate the cytotoxicity of the peptides on normal human cells. The cell lines were cultured as described previously [12].

The cell proliferation inhibitory rate was assessed by MTT assay as previously performed [12] with minor modifications. The peptide was diluted to a final concentration gradient from 1 µM to 100 µM, treating the cell lines for 24 h.

### 2.10. Lactate Dehydrogenase (LDH) Assay

The cell lines were cultured as previous section. After the cells were seeded in the 96-well plate for 12 h, different peptide doses were subjected to the cells for 6 h. The LDH released in the cell supernatant was detected using Pierce LDH cytotoxicity assay (Thermo Fisher Scientific, Loughborough, UK) [19].

## 3. Results

### 3.1. “Shotgun” Cloning of DPT9 Biosynthetic Precursor cDNA from a Skin Secretion-Derived cDNA Library of Phyllomedusa tarsius

**Figure 1:**
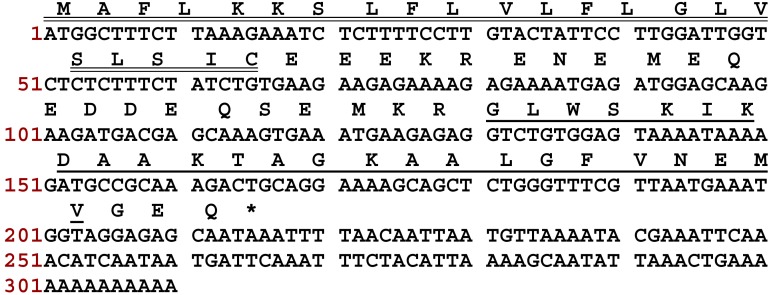
The nucleotide and translated open reading frame sequence of the precursor-encoding the cDNA cloned from the skin secretion of *Phyllomedusa tarsius*. The putative signal peptide is double-underlined. The mature peptide is single underlined for dermaseptin-PT9 (DPT9). The stop codon is indicated by an asterisk.

**Figure 2:**
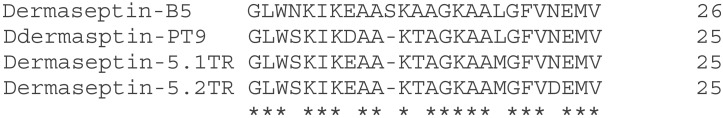
The alignments of DPT9 with selected dermaseptins. The asterisks indicate the identical amino acid residues in each sequence.

The full-length cDNA encoding the biosynthetic precursor of DPT9 was consistently cloned from the skin secretion-derived cDNA library of *Phyllomedusa tarsius*. The translated open reading frame consists of 71 residues (Figure 1). The biosynthetic precursor is constituted of a 22-residue putative signal peptide region, a Glu-rich acidic “spacer” domain that ends with a typical propeptide convertase processing site (-KR-), and a 25-residue mature peptide followed by an amidation consensus motif –GEQ-. The Gly residue in the C-terminus acted as an amide donor for posttranslational modification of mature peptide. Based on the result of BLAST analyses, DPT9 displayed high similarity to dermaseptin-5.1TR/5.2TR from *Phyllomedusa trinitatis* and dermaseptin-B5 from *Phyllomedusa bicolor* (Figure 2). The cDNA sequences of these dermaseptin precursors have been deposited in the GenBank Database under the accession codes MN399674.

### 3.2. Identification and Structural Characterization of DPT9

**Figure 3:**
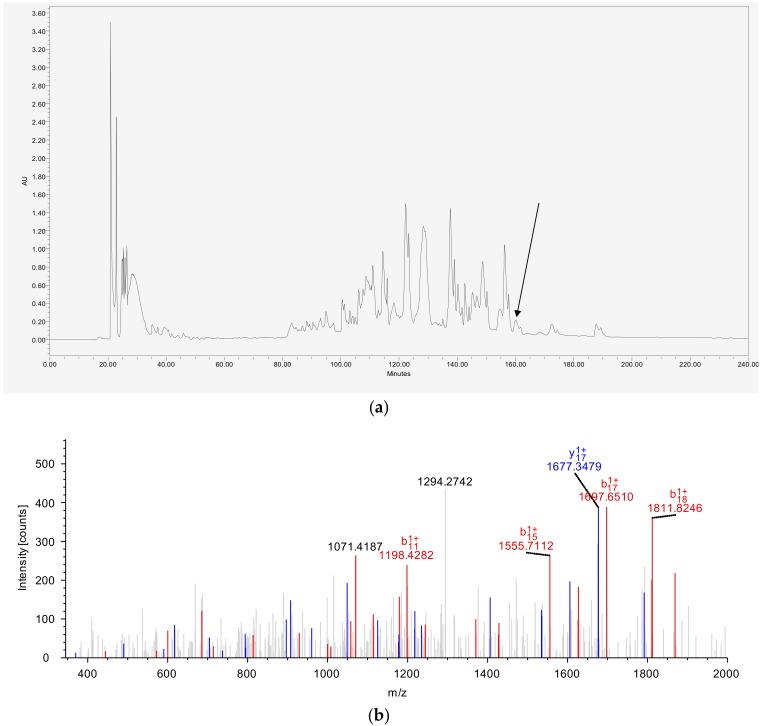
Identification of DPT9 from the corresponding skin secretions. (**a**) The retention time of DPT9 is indicated by an arrow in the HPLC chromatogram. (**b**) Tandem MS spectrum of DPT9 in the skin secretion. A doubly charged precursor ion (*m*/*z* 1302.62) was selected. The observed b and y ions are represented in red and blue, respectively.

The crude skin secretion of *Phyllomedusa tarsius* was analyzed by RP-HPLC (Figure 3a). The elution position of DPT9 is indicated in the chromatogram. The amino acid sequence of the mature peptide was further confirmed by LC-MS analysis against the translated proprepeptide sequence (Figure 3b). The post-translation modification of C-terminal amidation was also confirmed in the sequencing result, which corresponded to the motif of a glycine amidation present in the terminal position of the DPT9 precursor.

### 3.3. Synthesis, Predicted Physicochemical Properties and Secondary Structure of DPT9 and its Analogue K^8, 23^-DTP9

**Figure 4:**
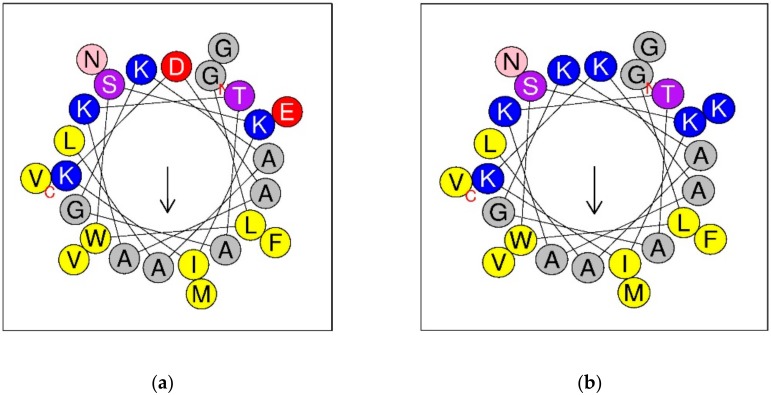
Helical wheel diagrams of DPT9 (**a**) and K^8, 23^-DPT9 (**b**). Positive charged residues are represented in blue circles, the negatively charged in red, the hydrophobic in yellow, the hydrophilic in purple, the amide in pink and the small residues in grey. Arrows indicate the direction of the hydrophobic moments.

**Figure 5:**
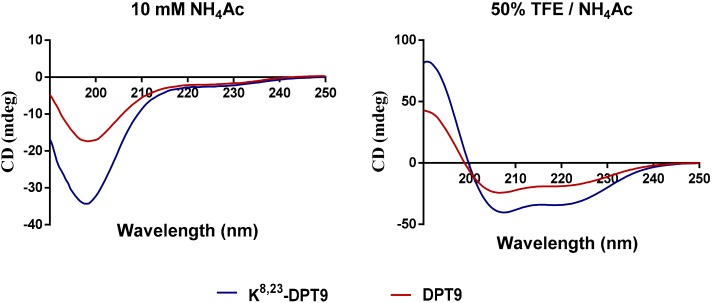
Circular dichroism (CD) spectra of 100 µM DPT9 (red line) and K^8, 23^-DPT9 (blue line) in 10 mM NH_4_Ac solution (pH 7.4) and in 50% trifluoroethanol (TFE)/NH_4_Ac solution (pH 7.4), respectively.

Helical wheel diagrams of DPT9 and its analogue, K^8, 23^-DTP9, showed they have the same direction of hydrophobic moment (Figure 4). Asp8 and Glu23 in the amino acid sequence of DPT9 were selected and substituted for Lys residues to increase the net charge from +2 to +6. Both DPT9 and K^8, 23^-DPT9 were chemically synthesized by solid phase Fmoc chemical method and purified by reversed phase HPLC (Figures S1 and S2). The predicted secondary structures of peptides were obtained using CD spectroscopy (Figure 5). The calculated helicity of the peptides was 24% and 39% using the DichroWeb server (Table 1). Besides, we compared the physico-chemical properties of both peptides with other demaseptins in our previous studies.

### 3.4. Antimicrobial and Haemolytic Activities of DPT9 and its Analogue K^8, 23^-DTP9

**Figure 6:**
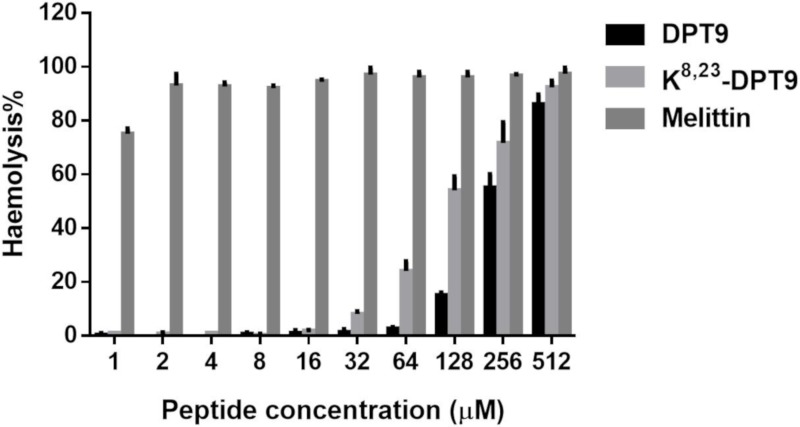
The haemolytic effects of DPT9, K^8, 23^-DPT9 and melittin towards horse red blood cells. The different peptide concentrations were compared to the positive control (100% haemolysis) where cells were incubated with 1% Triton X-100.

DPT9 and K^8, 23^-DPT9 exhibited broad-spectrum antimicrobial activity against pathogenic microorganisms including Gram-positive bacteria, *S. aureus*, MRSA and *E. faecalis*; Gram-negative bacteria, *E. coli*, *P. aeruginosa* and *K. pneumoniae*; the pathogenic yeast, *C. albicans* (Table 2). The antimicrobial activity of K^8, 23^-DPT9 was 16-fold more effective against MRSA and *C. albicans*, eight-fold more effective against *S. aureus* and *E. faecalis* and four-fold more effective against *E. coli*, *P. aeruginosa* and *K. pneumoniae*. DPT9 and its analogue were able to inhibit the biofilm formation of *S. aureus*, MRSA and *E. coli* (Table 3). K^8, 23^-DPT9 showed more effective anti-biofilm activity than DPT9. However, both peptides displayed weaker activity against biofilms that had already formed. Additionally, both peptides have low haemolysis on horse erythrocytes at the concentrations they effectively inhibit the microorganisms (Figure 6). The cationicity-enhanced analogue exhibited a stronger haemolytic effect than the parent peptide with the HC_50_ of DPT9 and K^8, 23^-DPT9 being 210 and 107 µM, respectively.

### 3.5. Membrane Permeabilisation of DPT9 and Its Analogue K^8, 23^-DTP9

**Figure 7:**
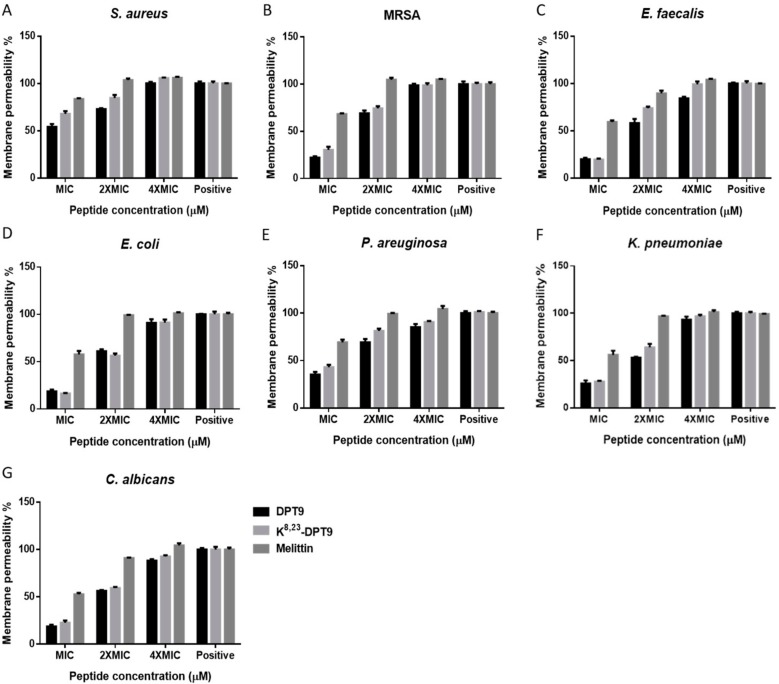
Membrane permeabilisation of DPT9, K^8, 23^-DPT9 and Melittin on *S. aureus* (**A**), MRSA (**B**), *E. faecalis* (**C**) *E. coli* (**D**), *P. aeruginosa* (**E**), *K. pneumoniaee* (**F**) and *C. albicans* (**G**) at peptide concentrations of MICs, 2 × MICs, and 4 × MICs. Positive control was obtained following incubation with 70% isopropyl alcohol. Data represent means ± SEM of five replicates.

Both peptides showed similar membrane permeabilisation effects on all the microorganisms (Figure 7). They demonstrated total, or close to total, membrane disruption at concentrations corresponding to four-fold the MICs, while moderate membrane permeabilization was observed at the MIC for each peptide.

### 3.6. MTT and LDH Assays on the Human Cancer and Normal Cells

**Figure 8:**
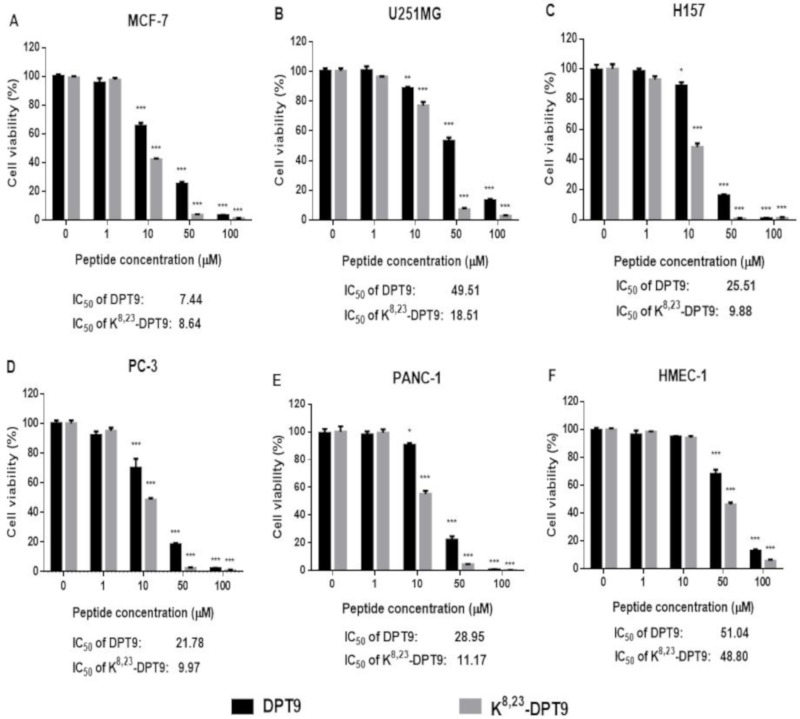
Cell viability of MCF-7 (**A**), U251MG (**B**), H157 (**C**), PC (**D**), PANC-1 (**E**) and HMEC-1 (**F**) after treatment with DPT9 and K^8, 23^-DPT9 for 24 h. The cell viability of growth control (without any treatment) was regarded as 100%. Data are represented as mean ± standard error of mean (SEM) with five replicates. The levels of significance are: * *p* < 0.05, ** *p* < 0.01, *** *p* < 0.001 by comparing all the concentrations to the growth control using one-way ANOVA.

DPT9 and K^8, 23^-DPT9 reduced the cell viability of all tested cell lines (Figure 8). K^8, 23^-DPT9 exhibited a more potent effect against the cancer cells than DPT9 with IC_50_s of 8.64~18.51 µM and 17.44~49.51 µM, respectively. However, both DPT9 and K^8, 23^-DPT9 were less effective on HMEC-1 with IC_50_ values of 51.04 µM and 48.85 µM, respectively.

**Figure 9:**
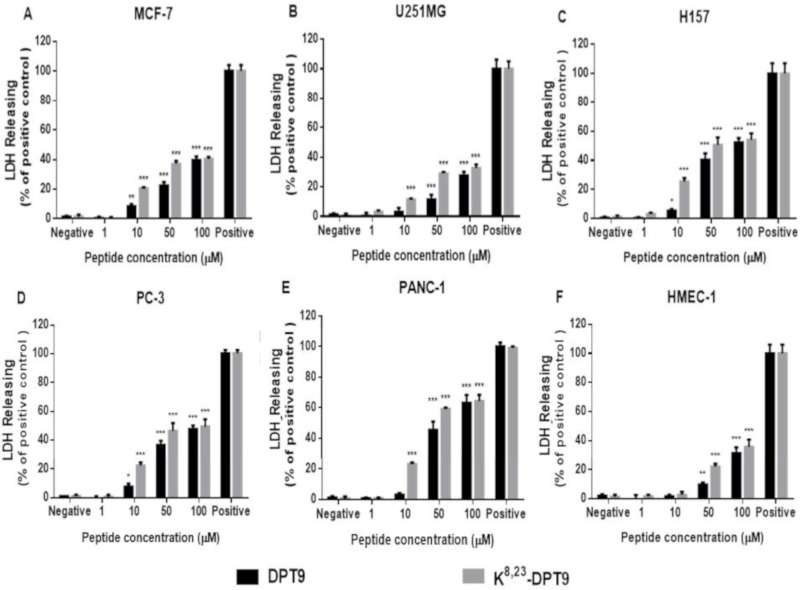
Lactate dehydrogenase (LDH) release from MCF-7 (**A**), U251MG (**B**), H157 (**C**), PC (**D**), PANC-1 (**E**) and HMEC-1 (**F**) after the treatment of DPT9 and K^8, 23^-DPT9 for 24 h. The treatment by 1× lysis buffer was indicated as positive control (100% cytotoxicity). The levels of significance are: * *p* < 0.05, ** *p* < 0.01, *** *p* < 0.001 by comparing all the concentrations to the negative control using one-way ANOVA.

Both peptides induced significant LDH releasing on different cell lines in 6 h. However, K^8, 23^-DPT9 showed more effective cytolysis on cancer cell membranes than DPT9 at concentrations ranging from 10 and 50 µM, which revealed that K^8, 23^-DPT9 were more effective at damaging cancer cell membranes (Figure 9). It is noteworthy that the cytotoxicity of the peptides at 100 µM on U251MG and MCF-7 were around 30% and 40%, respectively, but the cell viability of the peptides at the same concentration are less than 10%. Both peptides showed more effective ability of cell membrane damage to H157, PC-3 and PANC-1.

## 4. Discussion

Dermaseptin peptides have been studied since the 1990s and demonstrated to be a promising antimicrobial agent for the development of new therapeutic approaches [6]. To date, more than 100 dermaseptins have been identified from the skin secretion in the Uniprot database (access time: 05/10/2019), and their primary structures exhibited high degree of diversity. Herein, we discovered a novel dermaseptin, DPT9 from the skin secretion by the molecular cloning and LC-MS analysis. According to the alignments of the pre-propeptide, DPT9 shows structure motifs including a conserved region in the middle of the sequence and a Try residue typically present in position three [5,22]. The amino acid sequence is highly similar to the dermaseptin-5.1TR/5.2TR and dermaseptin-B5, possessing a N-terminal motif, GLWSKIK-, rather than the other motif, ALWKXXL/IL- [17]. So far, the biological functions of dermaseptin-5.1TR/5.2TR and dermaseptin-B5 have not been evaluated [17], we, therefore, initiated the study of DPT9 that could further help to deduce the bioactivity of dermaseptin-5.1TR/5.2TR and dermaseptin-B5. As results show, DPT9 has a broad range antimicrobial activity, with relatively low haemolytic activity on horse erythrocytes. It also showed more potent activity against Gram-negative bacteria, consistent with other dermaseptins [11,12,15]. 

Charge is one of main parameters for optimizing the bioactivity of antimicrobial peptides [23]. In our previous study, the cationicity enhanced analogue, K^5, 17^-DPS3 (+2 to +6), exhibited 4-32-fold potency against microbes [15]. In addition, K^5, 17^-DPS3, K^8, 23^-DPT9 was also designed by replacing two Lys and it exhibited similar fold change of antimicrobial activity. Furthermore, from the antimicrobial potency of the other dermaseptins in Table 2, it suggests that antimicrobial activity is significantly correlated with net charges to some extent. It is potentially due to the enhancement of the initial electrostatic attraction that is believed to contribute to the strong binding between cationic peptides and the anionic phospholipids and negatively-charged compounds (e.g., teichoic acid and lipopolysaccharide (LPS)) of bacteria [4,24].

Following initial membrane binding, the key event is the process of conformational transformation of the peptides [4]. When attaching on the lipid bilayer, dermaseptins could initiate the transformation to an α-helical structure, which is essential for their antimicrobial activity and membrane destabilization [25,26]. To our understanding, dermaseptins potentially form a helical structure at the N-terminal domain when interacting with lipids, and the amphipathcity and charges of this domain influence the biological activity with helix together [27]. K^8, 23^-DPT9 processes more helical content than DPT-9 in a membrane-mimetic environment. Although, the algorisms for calculating the helical contents of peptides via experimental CD spectra might not be precise enough, the helical contents of dermaseptins are not closely correlated with their activities as observed form Table 1 and Table 2. For instance, although DDU1 and DPS4 exhibit similar antimicrobial effects, the percentage of helix of DPS4 is larger than which of DDU1. Additionally, increasing helical content was observed when enhancing the cationicity of dermaseptins [15,27], which was indicated by the stablilisation of helix formation by charge-charge interaction and salt bridge [28].

Although it is proposed that membrane lysis can result via various mechanisms including membrane perforation, destruction, or solubilization, the microbicidal potential is thought to result from the capacity of the antimicrobial peptides to permeate the target cell membrane [9,29]. The hydrophobicity of α-helical antimicrobial peptides is a crucial feature for their interaction with the membrane bilayer and is required for effective permeability [30,31]. It has been demonstrated that dermaseptin B2 perturbs anionic bilayer membranes via a carpet-like model. The peptide residues at the hydrocarbon-water interface, and its hydrophobic portion is immersed in the hydrocarbon zone of the lipid bilayer. The interfacial location of dermaseptin B2 causes an asymmetric disturbance and induces a positive curvature in the bilayer, which results in the formation of transient pores and membrane permeation once the peptide accumulation reaches a threshold concentration [10].

In our previous study, increasing cationicity and hydrophobicity can enhance membranolytic activity, while hydrophibicity makes dermaseptin rather nonspecifically towards both bacterial and mammalian cell membrane [15]. It could enhance the interaction with lipids and its tendency to aggregate, which potentially exerts a “carpet model” [10]. On the other hand, owning more positive charges increased the binding affinity to the lipid bilayer, which allowed peptide accumulation on the membrane surface and facilitated the insertion capability by amphipathicity of peptide conformation [13,27]. With the same degree of amphipathicity and hydrophobicity, increasing net charges could be more specific to the negatively charged cells. For instance, Tat fusion for dermaseptin truncated derivative exhibited significant affinity to microorganisms rather than red blood cells [13]. DPT9 and K^8, 23^-DPT9 displayed significant membrane permeability at their two-fold MICs and four-fold MICs, which indicated that the peptides kill microorganisms by cell membrane disruption. However, in fact, K^8, 23^-DPT9 induced the same membrane permeability as DPT9 at lower concentration that is consistent with the consideration of enhanced binding affinity by net positive charges.

DPT9 and K^8, 23^-DPT9 also displayed anti-proliferative effects against tested human cancer cells. According to the results of LDH, both peptides could induce significantly release of LDH in 6 h against cancer cells, suggesting that the anticancer action of the peptides might be related to necrosis by cell membrane damage at the high concentration. Previous research has demonstrated that cancer cell membranes are generally negatively-charged, containing more phosphatidyserine and other anionic molecules including heparin sulphates and O-glycosylated mucins than normal cells [32,33]. Electrostatic interaction is believed to be a key factor for membrane binding between the cationic peptides and the cancer cell, and contribute to membrane lytic activity. It is, therefore, possible that K^8, 23^-DPT9 is more effective against cancer cell membranes and thus exert increased anticancer activity, comparing to DPT9. Whilst, both peptides could induce further cell death after 6 h when comparing the MTT and LDH results, indicating more mechanisms could be involved along with cell necrosis. Dermaseptin B2 can bind to the plasma membrane of human PC3 cancer cells, aggregate on and penetrate the cells, resulting in a necrotic-like pathway which ruptures the cancer cell membrane due to alteration in the mitochondrial membrane potential and stimulation of caspase-3 [34]. Furthermore, as previous data shows, dermaseptin-PS1 possessed cell membrane disruption result in cell necrosis at 10 μM, however, it up-regulated the expression of cleaved caspase 8 and activated the apoptotic pathway at lower concentrations [35]. Therefore, the anticancer action of both peptides could be also involved in both mechanisms among the different concentration ranges.

## Figures and Tables

**Table 1:** Physicochemical properties of DPT9 and K^8, 23^-DPT9.

	DPT9	K^8, 23^-DPT9	DPH ^a^	DCA1 ^b^	DDU1 ^b^	DPS3 ^c^	DPS4 ^d^
Molecular weight (Da)	2605.10	2617.24	2535.78	3024.62	2911.46	2550.00	2948.53
Hydrophobicity	0.348	0.326	0.333	0.291	0.331	0.373	0.368
Hydrophobic moment	0.447	0.462	0.427	0.263	0.255	0.437	0.226
Net charge	+2	+6	+1	+3	+3	+2	+3
α-helix (%)	24.18	46.14	35	25.3	27.9	44.9	67.6

DPH: ALWKEVLKNAGKAALNEINNLVQ-NH_2_ [12]. DCA1: ALWKDLLKNVGKAAGKAVLNKVTDMVNQ-NH_2_ [13]. DDU1: ALWKSLLKNVGKAAGKAALNAVTDMVNQ-NH_2_ [13]. DPS3: ALWKDILKNAGKAALNEINQIVQ-NH_2_ [15]. DPS4: ALWKTLLKHVGKAAGKAALNAVTDMVNQ-NH_2_ [16].

**Table 2:** The minimal inhibitory concentrations (MICs) and the minimal bactericidal concentrations (MBCs) of DPT9 and K^8, 23^-DPT9 against various microorganisms.

Microorganisms	MIC/MBC (µM)							
	MIC/MBC (µg/mL)							
	DPT9	K^8, 23^-DPT9	DPH	DCA1	DDU1	DPS3 ^a^	DPS4 ^a^	Melittin
*S. aureus NCTC 10788*	16/32	2/4	32/64	4/16	4/16	256	4	1/2
	41.7/83.4	5.2/10.4	81.1/162.2	12.1/48.4	11.6/46.4	652.5	11.8	2.8/5.7
Methicillin-resistant *Staphylococcus aureus* (MRSA) *NCTC 12493*	32/64	2/4	>512/>512	8/32	4/16	NA	8	1/4
	83.4/166.7	5.2/10.4	>1298.4/>1298.4	24.2/96.8	11.6/46.4		23.6	2.8/11.4
*E. faecalis NCTC 12697*	16/32	2/4	NA	128/256	64/128	NA	32	1/2
	41.7/83.4	5.2/10.4		387.1/774.1	185.6/371.2		94.3	2.8/5.7
*E. coli NCTC 10418*	8/16	2/4	16/16	4/16	4/16	8	8	2/4
	20.8/41.7	5.2/10.4	40.6/40.6	12.1/48.4	11.6/46.4	20.4	23.6	5.7/11.4
*P. aeruginosa ATCC 27853*	16/32	4/8	64>512	8/32	4/16	NA	16	16/32
	41.7/83.4	10.4/20.8	162.2/>1298.4	24.2/96.8	11.6/46.4		47.1	45.5/91.1
*K. pneumoniae ATCC 43816*	8/16	2/4	NA	8/128	4/64	NA	NA	2/8
	20.8/41.7	5.2/10.4		24.2/387.1	11.6/185.6			5.7/22.8
*C. albicans NCYC 1467*	64/128	4/8	16/64	4/16	4/16	4	4	2/4
	166.7/333.4	10.4/20.8	40.6/162.2	12.1/48.4	11.6/46.4	10.2	11.8	5.7/11.4

NA: Not tested in the previous studies. ^a^: MICs were only reported in the previous study.

**Table 3:** Antibiofilm activity of DPT9 and K^8, 23^-DPT9 against selected microorganisms.

Microorganisms	MBIC/MBEC (μM) ^a^	
	DPT9	K^8, 23^-DPT9
*S. aureus NCTC 10788*	16/32	2/4
*MRSA NCTC 12493*	32/64	2/4
*E. coli NCTC 10418*	8/16	2/4

^a^: MBIC: minimal biofilm inhibitory concentration. MBEC: minimal biofilm eradication concentration.

## Supplementary Materials

The following are available online at https://www.mdpi.com/2218-273X/9/10/628/s1, Figure S1: RP-HPLC chromatogram of purified DPT9 and K^8, 23^-DPT9. The acetonitrile gradient is indicated by dotted line. The purities of DPT9 and K^8, 23^-DPT9 were 97.5% and 96.3%, respectively. Figure S2: MALDI-TOF spectra of purified DPT9 and K^8, 23^-DPT9. The molecular weight of DPT9 and K^8, 23^-DPT9 were 2605.10 and 2617.24 Da, respectively. The observed mass to charge ratio m/z of [M + H]^+^ ions were 2605.49 and 2617.68, respectively.
