# Supplementary material for: A Novel Dermaseptin Isolated from the Skin Secretion of Phyllomedusa tarsius and Its Cationicity-Enhanced Analogue Exhibiting Effective Antimicrobial and Anti-Proliferative Activities
Source: Biomolecules. 2019 Oct 18;9(10):628. doi: 10.3390/biom9100628 (PMC6843903; doi:10.3390/biom9100628)
Supplement: Supplementary file 1 [file biomolecules-09-00628-s001.pdf]

A Novel Dermaseptin Isolated from the Skin Secretion of Phyllomedusa tarsius and its Cationicity-Enhanced Analogue Exhibiting Effective Antimicrobial and Anti-proliferative Activities

Supplement material

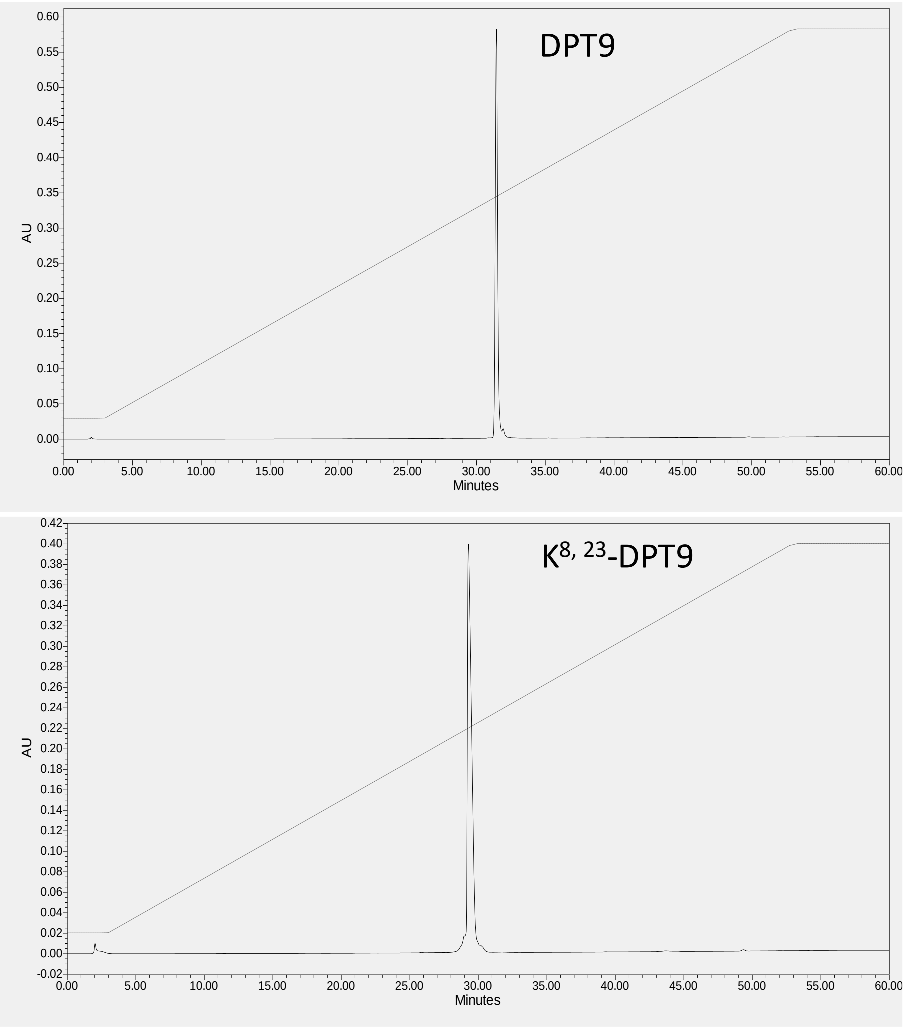

Figure S1. RP-HPLC chromatogram of purified DPT9 and $K^{8, 23}$-DPT9. The acetonitrile gradient is indicated by dotted line. The purities of DPT9 and $K^{8, 23}$-DPT9 were 97.5% and 96.3%, respectively.

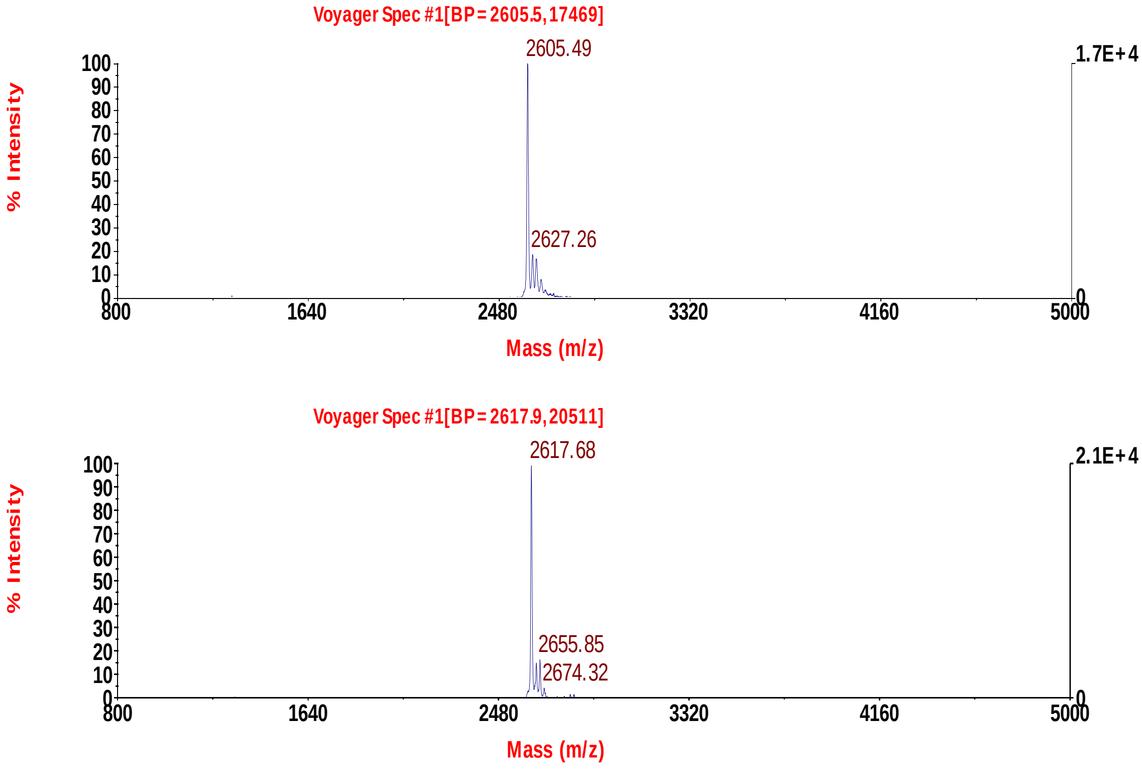

Figure S2. MALDI-TOF spectra of purified DPT9 and $K^{8,\,23}$-DPT9. The molecular weight of DPT9 and $K^{8,\,23}$-DPT9 were 2605.10 and 2617.24 Da, respectively. The observed mass to charge ratio m/z of $[M+H]^+$ ions were 2605.49 and 2617.68, respectively.
